# Genital Crohn's disease in pediatrics and genetic associations

**DOI:** 10.1002/jpr3.70022

**Published:** 2025-04-17

**Authors:** Erica Chang, Caroline Chinchilla Putzeys, Edward Hoffenberg, Ashish Patel, Elizabeth Hilow, Brad Pasternak

**Affiliations:** ^1^ Division of Pediatric Gastroenterology, Department of Pediatrics Children's Hospital of Philadelphia Philadelphia Pennsylvania USA; ^2^ Division of Pediatric Gastroenterology, Department of Pediatrics Phoenix Children's Phoenix Arizona USA; ^3^ Department of Pediatrics Digestive Health Institute, Children's Hospital Colorado Aurora Colorado USA

**Keywords:** inflammatory bowel disease, genetic variants, genital edema

## Abstract

Genital edema is a rare presentation of Crohn's disease (CD), also known as metastatic CD (MCD). This may precede, co‐occur with, or follow gastrointestinal symptoms and present a diagnostic challenge. We aimed to characterize the features, clinical courses, pathogenesis, and outcomes of patients with MCD to increase understanding and promote timely management. A retrospective review of four patients diagnosed with MCD was conducted at Phoenix Children's and Children's Hospital Colorado. Patients presented with painful and painless penoscrotal swelling with and without erythema. Scrotal histopathology revealed granulomatous inflammation, and genetic testing identified pathogenic variants in NOD2, COL7A1, and Chek2, as well as additional variants of uncertain significance. Treatments included antibiotics, steroids, biologics, and methotrexate with mixed responses. Further research and clinical trials are needed to better understand the pathogenesis and develop best practices.

## Abstract (highlights)

**What is Known:**

- Metastatic Crohn's disease (MCD) is a rare manifestation of CD that usually presents with genital swelling in pediatric patients.
- Knowledge regarding the pathogenesis of MCD is limited, and this atypical presentation may lead to delays in diagnosis and treatment.

**What is New:**

- Patients with MCD may have underlying genetic variants that play a role in pathogenesis.
- Our patients were found to have variants in NOD2, COL7A1, Chek2, and additional variants of uncertain significance.

## 1 INTRODUCTION

Metastatic Crohn's disease (MCD) is a cutaneous manifestation of Crohn's, which can present with non‐caseating granulomas in either genital or nongenital regions. In pediatrics, genital edema is typically seen and may precede gastrointestinal (GI) symptoms. This rare presentation has been described in several case reports to date and may present a diagnostic dilemma. Variable success has been reported with topical and systemic steroids, antibiotics, immunomodulators, and biologics. While NOD2 is known to play a key role in CD, the pathophysiology of MCD remains unclear. We describe four patients who presented with genital edema and were subsequently found to have clear or suspected CD. All patients were found to have pathogenic variants and variants of uncertain significance, which may play a role in the underlying disease process. Further research is needed to better understand the pathogenesis of MCD and to develop treatment guidelines.

## 2 METHODS

A retrospective chart review of four patients under 18 years old diagnosed with MCD was conducted between July 1, 2022 and June 12, 2024 at Phoenix Children's and Children's Hospital Colorado.

### 2.1 Ethics statement

Institutional Review Board approval was obtained at the respective sites, in addition to a Data Use Agreement at Children's Hospital of Philadelphia. Informed consent was not required for this study due to its exempt status.

## 3 CASE REPORT

### 3.1 Case 1

A 16‐year‐old uncircumcised male with growth hormone deficiency and Hashimoto's thyroiditis presented with 2 weeks of painless penoscrotal swelling. Approximately a year prior, he had two episodes that self‐resolved after a few days. GI symptoms were absent. Testicular ultrasound (U/S) showed possible phlegmon, and cephalexin was started. Fecal calprotectin was elevated, and endoscopy revealed antrum erythema, erythema/ulceration in the sigmoid, rectum, and anal canal, and a perianal skin tag. Biopsies showed chronic active gastritis, ileitis, and pancolitis with non‐necrotizing granulomata, and scrotal biopsy showed chronic lymphocytic dermatosis with granuloma. After not responding to corticosteroids, there was a good response to infliximab. However, he developed antibodies to infliximab and was transitioned to adalimumab. Despite appropriate adalimumab levels, he developed worsening colitis and was switched to risakinumab. Invitae Primary Immunodeficiency Panel identified a NOD2 variant c.2722G>C (p.Gly908Arg) and additional variants of uncertain significance in C1S, FANCI, FPR1, IGLL1, NFKBIA, POLE, and STIM1.

### 3.2 Case 2

3.2.1

A 10‐year‐old uncircumcised male with autism, Hashimoto's thyroiditis, eczema, and obesity developed painful penoscrotal edema with erythema. There was partial response over 1 year to topical steroids and multiple courses of cephalexin for presumed cellulitis. Testicular U/S indicated scrotal wall thickening and hyperemia. Due to frog‐spawn‐type papules along the medial foreskin, scrotal lymphedema was suspected; however, magnetic resonance angiography/magnetic resonance venography and lymphoscintigraphy were normal. Scrotal biopsy revealed non‐necrotizing granulomas. After identifying non‐bloody diarrhea, anal fissures, stooling accidents, reflux, abdominal pain, and urgency, endoscopy showed erythema, ulceration, and granularity at the sigmoid, ascending, and transverse colon. Biopsies showed gastritis, duodenitis, and pancolitis. He partially responded to adalimumab and prednisone with intermittent flares in the setting of low adalimumab levels. Invitae Inborn Errors of Immunity and Cytopenias Panel revealed a pathogenic variant in COL7A1 c.409C>T (p.Arg137) and additional variants of uncertain significance in NOD2, C3, CARD14, and NLRP12.

### 3.3 Case 3

A 5‐year‐old uncircumcised male with seasonal allergies developed a perianal rash that extended to the genitals over the course of a year and painless penile edema. This persisted despite multiple topical treatments and prednisone. Approximately 2 years after symptom onset, scrotal biopsy revealed granulomatous inflammation and dilated lymphatics. He also reported constipation and intermittent oral ulcers. Elevated fecal calprotectin led to endoscopic findings of ileocecal valve erythema, ulceration, and inflammation with anal fissure/skin tags. Biopsies showed duodenal villous blunting and intraepithelial lymphocytes, chronic inactive gastritis, chronic active ileitis, and non‐caseating granuloma at the ascending/descending colon. Circumcision was performed due to phimosis. Prednisone and infliximab were started. Celiac panel was positive, and he began a gluten‐free diet. Due to ongoing penile edema and undetectable infliximab levels, treatment was changed to a topical steroid, adalimumab, methotrexate, and prednisolone. Despite improvement in the perianal rash, his asymptomatic genital swelling persisted. Invitae Inborn Errors of Immunity and Cytopenias Panel revealed a pathogenic mutation in Chek2 c.846+4_846+7del and a variant of the uncertain significance of ANKZF1.

### 3.4 Case 4

A 4‐year‐old, previously healthy, circumcised male presented to the emergency department with swelling and erythema of the scrotum and penis for 6 months. He received cephalexin and topical hydrocortisone. U/S showed bilateral scrotal thickening with borderline hyperemia. After evaluation by urology, repeat U/S was recommended in 2 weeks, which was normal. Urinalysis and fecal calprotectin were also normal. He was subsequently evaluated by GI and denied GI symptoms. Endoscopic findings were visually normal, and biopsies showed mild esophagitis and mild chronic inactive gastritis. Video capsule showed grossly normal small bowel mucosa. Scrotal biopsies revealed non‐necrotizing granulomatous inflammation. Due to persistent edema, Invitae Inborn Errors of Immunity and Cytopenias Panel was obtained and indicated a heterozygous NOD2 variant c.2722G>C (p.Gly908Arg) and a variant of uncertain significance in TAPBP. Given the known association between NOD2 and inflammatory bowel disease (IBD), human leukocyte antigen testing was sent and pending ongoing discussions regarding potential tumor necrosis factor (TNF)‐α inhibitor initiation.

## 4 RESULTS

Three out of four patients presented with painless penile edema, and one patient reported associated pain. Most patients had additional autoimmune conditions such as Hashimoto's thyroiditis and celiac disease. Only two patients reported GI symptoms such as constipation, diarrhea, urgency, reflux, and occasional abdominal pain. Scrotal biopsy is key to diagnosis, and histopathology revealed non‐necrotizing granulomatous inflammation. Genetic testing identified pathogenic variants in NOD2, COL7A1, and Chek2 with additional variants of uncertain significance in C1S, FANCI, FPR1, IGLL1, NFKBIA, POLE, STIM1, NOD2, C3, CARD14, NLRP12, and ANKZF1 (Table 1). Most patients had notable delays in diagnosis and were treated for presumed infection (cellulitis and phlegmon). In addition to antibiotics, patients received topical and systemic steroids, infliximab, adalimumab, and methotrexate with mixed responses (Table 2).

**Table 1:** Pathogenic and variants of uncertain significance in patients.^a^

Genetic variant	Variant significance	Gene association
NOD2, c.2722G>C (p.Gly908Arg)	Increased risk allele	Numerous population‐based case–control studies have shown that this variant increases the risk of Crohn's disease
C1S, c.200A>G (p.Tyr67Cys)	Variant of uncertain significance^b^	C1S gene is associated with autosomal recessive C1S deficiency and autosomal dominant periodontal Ehler's Danlos syndrome
FPR1, c.556G>A (p.Asp186Asn)	Variant of uncertain significance	FPR1 gene currently has no well‐established disease association
IGLL1, c.594C>A (p.His198Gln)	Variant of uncertain significance	IGLL1 gene currently has no well‐established disease association
NFKBIA, c.473C>G (p.Ala158Gly)	Variant of uncertain significance	NFKBIA gene is associated with autosomal dominant anhidrotic EDA‐ID
POLE, c.3791G>T (p.Ser1264Ile)	Variant of uncertain significance	POLE gene is associated with autosomal dominant PPAP and autosomal recessive FILS syndrome
FANCI, c.3064G>T (p.Ala1022Ser) and c.754G>A(p.Glu252Lys)	Variants of uncertain significance	FANCI gene is associated with autosomal recessive FA‐I
STIM1, c.747G>T (p.Glu249Asp)	Variant of uncertain significance	STIM1 gene is associated with autosomal dominant TAM1, autosomal dominant STRMK syndrome, and autosomal recessive STIM1 deficiency
COL7A1, c.409C>T (P.Arg137*)	Pathogenic	COL7A1 gene is associated with autosomal DDEB and autosomal RDEB
NOD2, c.2546C>T (p.Ala849Val)	Variant of uncertain significance	This variant has been observed in individuals with Crohn's disease but is also present in population databases with an allele count higher than expected for a pathogenic variant
C3, c.3023C>T (p.Ser1008Leu)	Variant of uncertain significance	C3 gene is associated with autosomal recessive C3 deficiency, autosomal domain aHUS5, and autosomal dominant C3GN
CARD14, c.1918G>A (p.Val640Met)	Variant of uncertain significance	CARD14 gene is associated with autosomal dominant CMAPS (CARD14‐mediated psoriasis) and autosomal dominant pityriasis rubra pillaris
NLRP12, Exon 14 deletion	Variant of uncertain significance	NLRP12 gene is associated with autosomal domain FCAS
CHEK2: c.846+4_846+7del (intronic)	Pathogenic	CHEK2 gene is associated with autosomal dominant predisposition to breast, colon, thyroid, and prostate cancer
ANKZF1 c.1964G>A (p.Arg 655Gln)	Variant of uncertain significance	ANKZF1 gene currently has no well‐established disease association; however, there is preliminary evidence supporting a correlation with infantile‐onset inflammatory bowel disease
TAPBP, c.590C>T (p.Pro197Leu)	Variant of uncertain significance	TABP gene currently has no well‐established disease association; however, there is preliminary evidence supporting correlation with autosomal recessive hereditary MHC class I deficiency

Abbreviations: aHUS5, atypical hemolytic uremic syndrome 5; DDEB, dominant dystrophic epidermolysis bullosa; EDA‐ID, ectodermal dysplasia with T‐cell immunodeficiency; FA‐I, Fanconi anemia, type I; FCAS, familial cold autoinflammatory syndrome; FILS, facial dysmorphism, immunodeficiency, livedo, and short stature; MHC, major histocompatibility complex; RDEB, recessive dystrophic epidermolysis bullosa; STRMK, Stormorken; TAM1, tubular aggregate myopathy 1.

^a^ Information adapted from Invitae.^1^

^b^ Variants of uncertain significance currently do not have sufficient data and evidence to determine the effect of this variant.

**Table 2:** Summary of patients.

	Patient 1	Patient 2	Patient 3	Patient 4
Presentation age, sex	16, Male	10, Male	5, Male	4, Male
Past medical history	Growth hormone deficiency, untreated chronic lymphocytic thyroiditis	Autism, Hashimoto's, eczema, obesity	Seasonal allergies, Celiac disease	Previously healthy
Presenting symptoms	Painless penoscrotal swelling	Painful penoscrotal erythema and swelling	Perianal rash extending to genitals, painless penile edema	Painless penoscrotal swelling and erythema
Review of systems	Knee pain	Diarrhea Constipation Reflux Occasional abdominal pain Urgency with accidents	Oral ulcers Constipation	Foul smelling stools
Family history	No GI or autoimmune conditions	Mother with Hashimoto's, psoriasis, reflux, cholecystectomy	Sibling with oral ulcers, mother with oral ulcers and gluten intolerance	No GI or autoimmune conditions
Initial labs				
Albumin (g/dL)	3.4	4.4	3.6	3.8
Hgb (g/dL)	11.1	14.3	11.8	12.3
CRP (mg/dL)	1.0	5.6	<0.3	N/A
ESR (mm/h)	62	18	31	<3
Stool studies				
Fecal occult	Negative	N/A	N/A	N/A
Fecal calprotectin (µg/g)	547	374	639	27.2
Stool PCR	Negative	N/A	N/A	N/A
Imaging	U/S possible phlegmon/developing abscess without torsion CTE inflammatory changes in terminal ileum and rectosigmoid colon, perianal fistula	U/S scrotal wall thickening with echogenic fat in scrotum without torsion MRI pelvis (diffuse scrotal wall edema), MRA/MRV normal Lymphoscintigraphy normal	MRE terminal ileum mucosal irregularity and bowel wall thickening	U/S bilateral scrotal skin thickening with borderline hyperemia without torsion CT chest/abdomen/pelvis normal
EGD/colonoscopy	Gastritis, erythema, and ulceration at rectosigmoid colon and anal canal	Erythema and ulceration at sigmoid, ascending, and transverse colon	Ileocecal valve erythema/ulceration, anal fissure, skin tags	Normal
Histopathology	Gastric granuloma, ileitis/pancolitis with non‐necrotizing granulomas	Gastritis with reactive changes in esophagus, chronic duodenitis, active colitis at cecum, ascending, transverse, and descending colon with chronic colitis at rectosigmoid	Duodenal villous blunting, crypt hyperplasia, mild lamina propria lymphoplasmacytoid, intraepithelial lymphocytosis, moderate active ileitis, ileocolonic intraepithelial neutrophils, ascending and descending colon granulomata, multinucleated giant cells with associated lamina propria calcification/foreign body in descending colon	Mild esophagitis with rare intraepithelial eosinophils, mild chronic inactive gastritis
Scrotal biopsy	Chronic lymphocytic dermatosis with granuloma	Non‐necrotizing granulomas and no evidence of lymphatic malfunction	Granulomatous inflammation, dilated lymphatics, scattered polarizable foreign material	Non‐necrotizing granulomatous inflammation
Genetic variants	NOD2 variant c.2722G>C (p.Gly908Arg) Additional variants of uncertain significance in C1S, FANCI, FPR1, IGLL1, NFKBIA, POLE, STIM1	COL7A1 variant c.409C>T (p.Arg137) Additional variant of uncertain significance in NOD2, C3, CARD14, NLRP12	Chek 2: c.846+4_846+7del (intronic) Additional variant of uncertain significance in ANKZF1	NOD2 variant c.2722G>C (p.Gly908Arg) Additional variant of uncertain significance in TAPBP
Treatments before biologics	Antibiotics	Antibiotics, topical steroids and diphenhydramine	Multiple topicals, oral steroids	Antibiotics, topical steroids
Initial biologic	Infliximab with resolution of swelling but developed antibodies, switched to Adalimumab; developed worsening colitis and now on Risakinumab	Adalimumab with worsening swelling and erythema a few months later in the setting of low levels increased dosing to q1wk	Infliximab without improvement in symptoms and developed antibodies, switched to Adalimumab and methotrexate	Ongoing discussions regarding potential anti‐TNF initiation
Current therapy	Risakinumab (monitoring response)	Adalimumab (monitoring response)	Adalimumab and methotrexate (improvement in perianal rash only)	N/A

Abbreviations: CRP, C‐reactive protein; CT, computer tomography; CTE, computer tomography enterography; EGD, esophagogastroduodenoscopy; ESR, erythrocyte sedimentation rate; GI, gastrointestinal; Hgb, hemoglobin; MRA, magnetic resonance angiography; MRE, magnetic resonance elastography; MRV, magnetic resonance venography; PCR, polymerase chain reaction; TNF, tumor necrosis factor; U/S, ultrasound.

## 5 DISCUSSION

CD is an IBD that may involve any part of the GI tract. Extraintestinal manifestations (EIMs) are also common with approximately 6%–47% of patients with IBD experiencing at least one EIM.^2^ This may include arthralgia/arthritis, aphthous stomatitis, primary sclerosing cholangitis, pancreatitis, iritis/uveitis, ankylosing spondylitis, and nephrolithiasis.^3^ Cutaneous manifestations can also occur in a minority of patients, most commonly erythema nodosum and pyoderma gangrenosum.^4^ On rare occasions, there can also be noncontiguous dermatological spread of CD involving the genitalia and perineum. This condition is known as MCD, which was first described by Parks et al. in 1965.^5^

**Figure 1:**
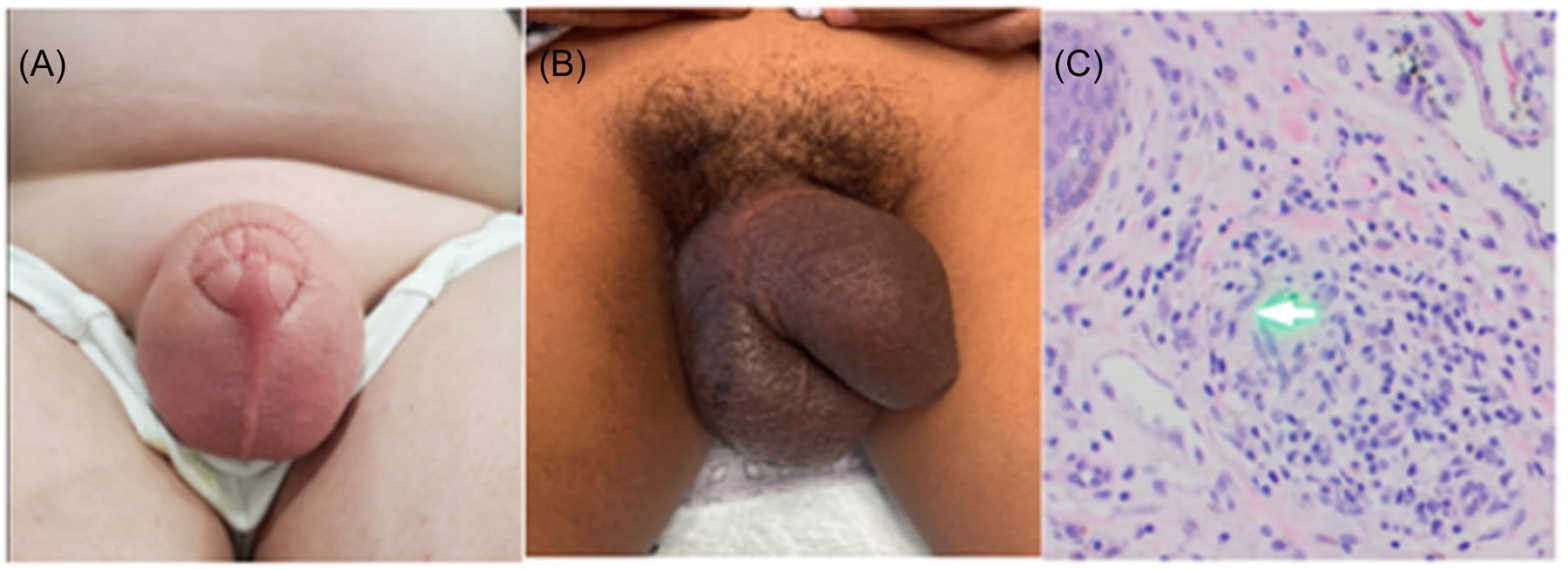
Features of metastatic Crohn's disease. (A) Penoscrotal swelling with erythema, (B) penoscrotal swelling without erythema, and (C) chronic lymphocytic dermatosis with granuloma of scrotum.

Less than 100 cases of pediatric MCD have been reported in the literature to date.^6^ These lesions are characterized by swelling, plaques, nodules, fissures, ulcerations, or crusts.^4^, ^7^, ^8^ In children, MCD typically presents as genital swelling with or without erythema in approximately 85% of cases (Figure 1).^4^ The mean age of onset is 11.1 years (range: 5–17 years), and the incidence in both males and females is reported to be equal.^6^ Prior studies have shown that MCD co‐occurs with CD in 50.8% of children, while others may develop GI symptoms after MCD diagnosis (15.3%) or even lack signs of CD (11.9%).^6^, ^7^ While there is no correlation reported with the severity of intestinal disease, MCD is more common in patients with colonic disease.^9^

The diagnosis of MCD is challenging and may be confused with cellulitis, sexually transmitted infections, trauma, erysipelas, vasculitis (Behcet's), hidradenitis suppurativa, mevalonate kinase deficiency, and allergic dermatitis.^10^, ^11^, ^12^ As a result, biopsy of the affected area is key to diagnosis. On histopathology, MCD is characterized by noncaseating granulomas and may also include granulomatous lymphangitis. Upon identifying granulomatous inflammation, other etiologies should be considered, such as sarcoidosis, infections (mycobacterial or fungal), and foreign body‐related inflammation.^7^, ^12^ MCD may be especially challenging to differentiate from sarcoidosis, but infiltrate with numerous eosinophils, ulcerated epidermis, and significant dermis edema are not found in sarcoidosis and point towards MCD.^13^

The pathogenesis of MCD is unclear. Various mechanisms have been proposed, such as circulating immune complexes, type IV hypersensitivity reaction, a multifactorial process involving the immune system, alteration of enzymes, and genetic risk factors.^5^, ^14^ All four of our patients were found to have pathogenic and unknown genetic variants (Table 2), including NOD2 which has a known association with CD. Although various loci have also been found to be associated with cutaneous EIMs, specific variants for MCD have not been identified.^3^ To our knowledge, this is the first report of genetic variants in MCD patients. The pathogenic variants that were identified include NOD2 c.2722G>C (p.Gly908Arg), COL7A1 c.409C>T (p.Arg137*), and CHEK2 c.846+4_846+7del (intronic). For this specific NOD2 variant, studies have shown that this change leads to decreased NFkB activity and decreased response to lipopolysaccharide and peptidoglycan.^15^, ^16^ However, much remains unknown regarding NOD2's complex role, and it may be possible that there is a phenotypic association with genital edema and erythema. COL7A1 gene encodes the alpha‐1 chain of type VII collagen, and this specific variant is associated with autosomal dominant and recessive dystrophic epidermolysis bullosa. Studies have shown an association between CD and autoantibodies against type VII collagen, which exists in the basement membrane of stratified squamous epithelia.^17^ While this may lead to cutaneous manifestations, type VII collagen plays a role in maintaining cell–matrix adhesion, and its dysfunction typically manifests as blisters. Although unclear, it is possible that disruption of the epithelial barrier may increase predisposition to MCD. Finally, CHEK2 is a tumor suppressor gene, and variants are associated with cancers such as thyroid, prostate, and colorectal cancer.^18^ Thus, while this gene may lead to GI manifestations in the form of malignancy, its role in MCD is less certain. Additional variants were also identified in C1S, FANCI, FPR1, IGLL1, NFKBIA, POLE, STIM1, NOD2, C3, CARD14, NLRP12, and ANKZF1; however, insufficient data exists in the literature to determine the effect of these changes (Table 1).^1^

Interestingly, our patients were also found to have additional conditions such as celiac disease and Hashimoto thyroiditis, which suggests that genital MCD may be linked with other autoimmunity. While a specific correlation between genital Crohn's and autoimmune conditions has not been established, the overlap of CD and immune‐mediated diseases (such as celiac disease and hypothyroidism) has been previously reported in the literature.^19^, ^20^, ^21^ Based on current knowledge, these processes likely involve environmental triggers in individuals with genetic predisposition leading to immune dysregulation and inflammation.

Treatment of MCD may include antibiotics, aminosalicylates, topical/systemic steroids, immunomodulators, biologics, or surgical interventions. However, individual responses to therapy are highly variable, and consensus guidelines for treatment are lacking. Prior case reports and case series have shown positive responses to TNF‐α inhibitors, but relapses may be common. Similarly, only partial improvement was noted in our patients treated with infliximab and adalimumab. Further research and randomized controlled studies are needed to establish effective treatment guidelines and improve patient outcomes.

Our cases demonstrate the importance of keeping a high index of suspicion for CD and genetic abnormalities in patients presenting with genital lesions without clear cause. Many patients do not demonstrate GI symptoms and may experience significant delays in diagnosis. Thus, increasing awareness can help promote timely diagnosis and management. Additional studies are needed to elucidate pathogenesis and develop effective treatment protocols.

## CONFLICT OF INTEREST STATEMENT

The authors declare no conflicts of interest.
